# Fertilisation Success in *Acanthaster* cf. *solaris* and *Acanthaster brevispinus* and Reciprocal Hybrid Crosses Across a Range of Sperm Concentrations and Development to the Hybrid Juvenile

**DOI:** 10.1002/ece3.74560

**Published:** 2026-11-06

**Authors:** Ronan T. Hill, Matthew Clements, Monique Webb, Brigitte Sommer, Maria Byrne

**Affiliations:** ^1^ University of Sydney Camperdown New South Wales Australia

**Keywords:** COTS, high‐latitude reefs, hybridisation, reproductive, species introgression

## Abstract

Novel species interactions between resident and range shifting species in response to warming are posing challenges for conservation and management. This is especially the case when major predatory species are involved, as seen in the appearance of a mixed species outbreak of coral‐eating crown‐of‐thorns sea stars *Acanthaster* cf. *solaris* (As) and the lesser‐known *Acanthaster brevispinus* (Ab) in the tropical to subtropical transition zone in eastern Australia. The outbreak consisted of reproductively mature animals, and we investigated the potential for cross‐species hybridisation in fertilisation experiments across a range of sperm concentrations to sperm limitation conditions (10^6^–10^2^ sperm mL^−1^). Fertilisation was successful in all combinations of parents (As ♂ × As ♀, Ab ♂ × Ab ♀, Ab ♀ × As ♂, As ♀ × Ab ♂) with successful hybrid crosses in sperm dilutions as low as 10^2^ sperm ml^−1^. Development in conspecific and heterospecific crosses was similar through the bipinnaria and brachiolaria larval stages to the juvenile. Juvenile hybrids had typical juvenile *Acanthaster* spp. development with a stellate five‐armed profile post metamorphosis, gaining arms after ~78 days. At no stage did the juvenile hybrids develop the distinctive pink colour trait that is characteristic of *Acanthaster* cf. *solaris* juveniles. Our results indicate that hybridisation is not restricted by sperm limitation conditions, with subsequent development of hybrids to the advanced juvenile stage. Our results highlight the potential for hybridisation and species introgression when range shifting species overlap with sister taxa. This may facilitate further range expansion or development of resilient hybrid progeny as a risk to vulnerable high latitude coral communities.

## Graphical abstract

Recent appearance of a mixed species outbreak of coral‐eating crown‐of‐thorns sea stars *Acanthaster* cf. *solaris* and the lesser‐known *Acanthaster brevispinus* in the tropical to subtropical transition zone in eastern Australia have caused management concern due to the potential of hybridisation. We investigated the potential for cross‐species hybridisation in fertilisation experiments across a range of sperm concentrations to sperm limitation conditions and subsequent development to the juvenile. Fertilisation was successful in all combinations of parents, with successful development in hybrids to the juvenile stage.

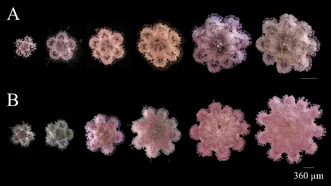

## 1 Introduction

Climate change poses challenges for conservation and management as species are undergoing poleward range extension, particularly in marine regions with strong boundary currents (Zarzyczny et al. 2024; Sunday et al. 2011; Stuart‐Smith et al. 2017). This is especially prominent in subtropical and temperate regions as warm affinity organisms are shifting poleward. This is generating new interactions between resident and shifting species that can alter trophic and ecosystem function (Vergés et al. 2019). The impacts of species range shift are especially severe when ecologically important species such as habitat‐formers (e.g., coral, macroalgae) and their predators/grazers are involved (Vergés et al. 2014; Cooney et al. 2024; Wernberg et al. 2024; Sommer et al. 2025). Benthic communities in subtropical and temperate regions are experiencing changes in species abundance and composition due to warming and increased abundance and poleward shifts of tropical herbivorous fishes and invertebrates, as well as coral predators that pose as a threat to these reefs (Vergés et al. 2016; Yasuda 2018; Sommer et al. 2025).

When species shift to areas where closely related sister taxa are present there is potential for hybridisation, leading to introgression, fusion of species or hybrid speciation (Mallet 2005, 2007; Baack and Rieseberg 2007). If sister taxa are reproductively compatible, then introgression in the new overlapping portion of their range can drive biodiversity loss or potential novelty in speciation (reticulate evolution) (Bronstein et al. 2019). Hybridisation can facilitate further range expansion through increased genetic variation and adaptative evolution at the range edge (Pfenning et al. 2016), and generation of resilient hybrid progeny/species (Mallet 2007; Baack and Rieseberg 2007; Arnold and Fogarty 2009; Wang et al. 2024). Hybridisation and introgression have been reported for several echinoderms; including sea stars (*Meridiastra*: Byrne and Anderson 1994; *Asterias*: Harper and Hart 2005, 2007), brittle stars (*Ophioderma*: Weber et al. 2019) and sea urchins (*Tripneustes*: Bronstein et al. 2016, 2017; McLaren et al. 2023) (*Strongylocentrotus*: Glasenapp and Pogson 2023). The integration of gene pools can lead to different morphological characteristics and integration of genetic material, especially in advanced‐generation hybrids (Harper and Hart 2007; Arnold and Fogarty 2009).

The impacts of species' range shifts are seen in the appearance of a mixed species outbreak of coral‐eating crown‐of‐thorns sea stars (COTS) *Acanthaster* cf. *solaris* and the lesser‐known *Acanthaster brevispinus* in subtropical eastern Australia (Sommer et al. 2025). The west Pacific species *Acanthaster* cf. *solaris* is abundant on shallow water coral reefs where outbreaks can cause significant coral decline (Deaker and Byrne 2022; Foo et al. 2024). The short‐spined *Acanthaster brevispinus* has an Indo‐Pacific distribution and is most commonly found in deeper (63 m) sediment/muddy habitats where it preys on a range of invertebrates including soft corals and solitary hard corals (Fisher 1917; Birkeland and Lucas 1990; Endean 1961; Yasua et al. 2017; Keesing et al. 2023). Although the feeding biology of *A. brevispinus* is not well understood in the presence of abundant coral, this species can be a major coral predator (Keesing et al. 2023; Sommer et al. 2025).

Mixed species outbreaks of the two COTS species on shallow, high‐latitude reefs are unusual with two reports in eastern Australia and Japan (Yasuda 2018; Sommer et al. 2025). These reefs have been considered as a refuge for tropical species under ocean warming (Beger et al. 2014) and support diverse coral assemblages including tropical species at their poleward range limits, as well as high‐latitude specialists and small‐range endemics (Mizerek et al. 2016; Malcolm et al. 2026). In the Solitary Islands Marine Park (SIMP) in eastern Australia (30° S), a mixed aggregation of the two COTS species was seen preying on a broad range of corals, including endemic species and many slow growing massive corals (Sommer et al. 2025). As a result, management conducted a culling operation to remove these sea stars as a means to protect the high conservation value of these corals in the park (Figure 1) (Sommer et al. 2025).

**FIGURE 1:**
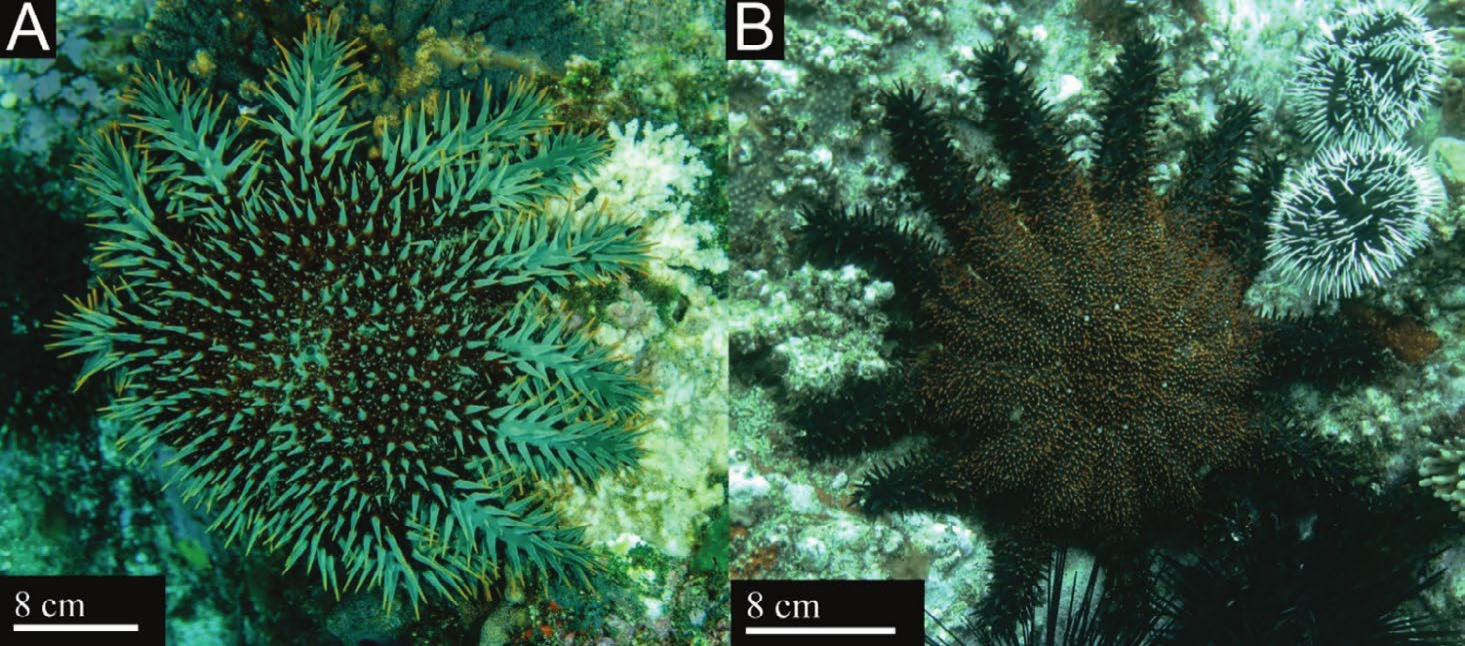
*Acanthaste*r in the Solitary Islands Marine Park. (A) Adult *Acanthaste*r cf. *solaris* on tabular *Acropora* coral; (B) Adult *Acanthaster brevispinus* on benthic surface next to *Tripneustes australiae* (above) and *Diadema savignyi* (below). Photo of *Acanthaste*r cf. *solaris* courtesy of Ian Shaw.

Understanding the interactions of these two *Acanthaster* species in their new overlap range is important due to the major threat these coral predators pose to coral reef ecosystems. This is especially important as these two species can hybridise and the hybrids are viable to sexual maturity (Lucas and Jones 1976; Lucas 1984). Although it has been shown that these two species can hybridise (Lucas and Jones 1976), there is no research on fertilisation success under sperm limitation conditions and limited research on the subsequent developmental stages. Previous studies on the hybrids between *Acanthaster* cf. *solaris* and *Acanthaster brevispinus* show that juvenile development is similar to that documented for *A*. cf. *solaris* (Lucas and Jones 1976; Lucas 1984). Hybridisation between the two COTS species is also noted to result in abnormal or failed juvenile development (Lucas and Jones 1976).

Sperm limitation through dilution effects is a major restrictive factor for successful fertilisation of marine species in nature (Benzie and Dixon 1994; Levitan 1998; Levitan et al. 1991; Levitan and Petersen 1995). This has been shown in COTS, where fertilisation success is influenced by sperm limiting conditions, gamete age and time of breeding season (Benzie and Dixon 1994). Fertilisation rates decreased with gamete age, with 50% fertilisation achieved with gametes used 1 h after collection compared with > 90% fertilisation success when using fresh gametes in sperm concentrations as low as 10^3^ sperm mL^−1^ (Benzie and Dixon 1994).

In echinoderms, egg size variability within species and between females also affects the target size of the egg for spermatozoa (Levitan 1993). Larger eggs have a higher likelihood of fertilisation success, especially in sperm limitation conditions (Levitan 1998; Farley and Levitan 2001). The eggs of *Acanthaster* cf. *solaris* are larger than those of other planktotrophic larvae (~250 μm) (Caballes et al. 2016), which likely facilitates a higher fertilisation success under sperm limitation conditions due to a large egg target size for sperm (Farley and Levitan 2001; Podolsky 2001; Levitan 2006). The egg size of *A. brevispinus* is not known.

In this study we investigated the fertilisation biology of the two *Acanthaster* spp. and the potential for hybridisation across a range of sperm concentrations, including what may be limitation conditions. Cultures were established to compare development in conspecific and heterospecific crosses.

## 2 Methods

### 2.1 Species Collection and Maintenance

*Acanthaster brevispinus* (Ab) were collected from SIMP in New South Wales (29.92° N, 153.38° E) and transported to Sydney Institute of Marine Science (SIMS) in Feb 2025 maintained in flow‐through aquaria supplied with filtered seawater (FSW 5.0 μm) at 24°C, the temperature near the collection site. The taxonomy of the west Pacific species of COTS is uncertain due to a lack of type specimen and clear type locality (Haszprunar and Spies 2014; Hutchings et al. 2025). Here, we apply the current name in use for this COTS, *Acanthaster* cf. *solaris*. Mature *A*. cf. *solaris* (As) were collected from the Northern Great Barrier Reef by the COTS control team in November and December 2024 and were shipped to the SIMS where they were maintained in flow‐through aquaria supplied with filtered seawater (FSW 5.0 μm) at 26°C, the temperature near the collection site. Gender of each specimen was determined by gonad biopsy through an incision at the base of the arms.

*Acanthaster brevispinus* is well known to be morphologically and molecularly distinct from the west Pacific species of COTS (Vogler et al. 2008; Yasuda et al. 2006). The taxonomic status of the *A. brevispinus* from the study population was confirmed based on morphological traits and genetic analyses of three individuals collected from SIMP (Sommer et al. 2025).

### 2.2 Egg Size and Fertilisation Assay

All experiments were conducted in a controlled temperature room (25°C–26°C), with 1‐μm filtered fresh sea water (FSW), salinity at 34 ppt. Fertilisations were conducted using eggs from individual females and sperm pooled from two males, with the number of fertilisations per cross type determined by availability of females. As all crosses used one female and two males with three cultures per fertilisation, each culture represents a distinct embryo genotype pool. The species crosses were done in all combinations of egg and sperm of the two species (cross code—genus and species): controls As ♂ × As ♀ (2 fertilisations—6 replicate embryo genotype pools) and Ab ♂ × Ab ♀ (2 fertilisations—6 replicate embryo genotype pools); As ♀ × Ab ♂ (1 fertilisation—3 replicate embryo genotype pools) and Ab ♀ × As ♂ (1 fertilisation—3 replicate embryo genotype pools). As we only had one mature female for both *Acanthaster* cf. *solaris* and *Acanthaster brevispinus* available for this study, each hybrid cross was from 1 female and 2 males.

To obtain gametes, small pieces of ovary and testes were removed after making a small incision in the body wall. Ovaries were rinsed with FSW and placed in 10^−5^ M 1‐methyl adenine in FSW to induce ovulation. After 30 min, the eggs were collected and placed in 100 mL of FSW. Eggs were checked under a microscope for quality and germinal vesicle breakdown. The total number of eggs for each treatment was measured from a 100 mL suspension through counts of 200 μL aliquots. A sample of eggs from each female was photographed for later measurement to assess for potential differences in egg target size for sperm using an Olympus compound microscope with an attached DP23 digital microscope camera and Olympus CellSens software.

Sperm was collected directly from the testes placed in small tubes and stored at 4°C. A small sample of sperm from two males of the same species was combined and macerated in a small petri dish. Sperm was checked microscopically for motility and then promptly used in fertilisation. The density of sperm in 10 μL of the semen sample was determined using a haemocytometer and this calculation determined how much sperm to add to 50 mL of FSW to create a stock solution at 10^7^ sperm/mL. A small sample (1 mL) of the stock was placed into a small tube and stored at 4°C to do a sperm count. The sperm dilution gradient was created by diluting the concentrated stock solution through a series of 10‐fold dilutions giving a range of sperm dilutions from 10^6^ to 10^2^ sperm mL^−1^ in 50 mL of FSW.

For each fertilisation assay 200 eggs were placed in a small (140 mL) 80 μm mesh filter basket (3 per sperm concentration) in FSW in a six well plate. As we had 5 sperm concentration treatments (10^6^–10^2^) a total of 15 baskets were prepared for each run of the experiment. For two rinses we prepared glass Petri dishes filled with FSW for rinse one and a new 6‐well dish with 10 mL of FSW in each well for rinse two. For fertilisation the egg baskets were simultaneously placed into the relative sperm dilution for 10 s (see Styan et al. 2005). The duration of exposure of eggs to sperm was consistent as timed by a stopwatch. After 10 s, all egg baskets were simultaneously removed using several pairs of hands and placed into the Petri dishes (rinse 1) and then into the wells of the six‐well plate (rinse 2). The transfer between the two rinses took approximately 15 s. After 30 min, the eggs from each basket were photographed (as above). Fertilisation success was determined by the presence of a fertilisation envelope (*n* = 100 eggs scored per photograph).

### 2.3 Spawning and Rearing of Larvae and Juveniles

A separate set of fertilisations were done for each cross type to establish larval cultures following routine methods (Clements et al. 2022). The embryos were reared for 24 h at 25°C–26°C in 5 L beakers of 1 μm FSW to the gastrula stage at which time the water was changed, and gastrula were transferred to 1 L beakers. The cultures were gently aerated. Larval density in each beaker was approximately 10 larvae per ml. When the bipinnaria stage was reached the larvae were fed with 7:3 ratio of *Tisochrysis lutea* and *Isochyrsis galbana* (10,000 cells mL^−1^) every 2 days following water renewal using reverse aspiration. The bipinnaria (7 days post fertilisation: dpf) and brachiolaria (21 dpf) stage larvae were photographed (as above). Larvae from all crosses were measured for larval length, width, surface area and ciliated band length. Larvae were sampled randomly across two to three independent cultures established for each cross.

For all cross types, competent brachiolaria larvae were identified microscopically by the presence of an attachment complex and hand‐selected for induction of metamorphosis. While there was some variation in larval development within cultures, brachiolaria appeared at approximately the same time to allow selection of competent larvae for parallel settlement trials using all crosses. The larvae were transferred into 6‐well plates with 1 μm FSW at 26°C and a settlement inducer, a small piece of coralline alga (*Amphiroa* sp.). This alga induces metamorphosis and serves as a food source for the *Acanthaster* cf. *solaris* juveniles (Deaker et al. 2021). No Ab ♂ × Ab ♀ brachiolaria settled in the presence of *Amphiroa*. The juveniles were fed fresh *Amphiroa* sp. every 3 days with parallel water changes. They were photographed (as above) and measured for diameter (arm tip to arm tip), surface area, central disk diameter and arm length as well as number of arms. Juveniles were measured at settlement (*n* = 10), 5 days post metamorphosis (dpm) (*n* = 13), 30 dpm (*n* = 11) and 78 dpm (*n* = 12). Juveniles continued to live beyond 250 dpm when observations ceased. The images of the larvae and juveniles were analysed using ImageJ software (v1.54g).

### 2.4 Statistical Analyses

To estimate the relationships between proportional fertilisation success, sperm concentration and species cross, binomial generalised linear mixed models (GLMMS) were applied, with sperm concentration treated as a continuous numeric predictor, and fertilisation treatment as a categorical predictor. Observation number was included as a random effect to account for differences in treatment handling. Fertilisation success was examined as proportional data binding the number of successes and failures (i.e., fertilised eggs and unfertilised eggs). The *glmmTMB* function was used for models from the glmmTMB package (Brooks et al. 2017).

Egg diameter data for both species were analysed using a linear mixed effects model. Diameter was treated as a continuous numeric predictor and species as a categorical predictor. Individual females were added as random effects.

Data for all larval bipinnaria size measurements were analysed using a 2‐way analysis of variance (ANOVA), with hybridisation treatment as the fixed factor. These tests were performed to determine how morphological traits (length, width, surface area and ciliated band length) differed between hybrid type at the end of development from maternal provisions. Post hoc comparisons of the data were analysed using Tukey's HSD test to determine differences between treatments.

Hybrid juvenile size data over ~2 months post settlement were analysed using a multivariate analysis of variance (MANOVA) to test for the effects of hybridisation type and days post metamorphosis with their interaction on juvenile morphological traits. The response variables included diameter, surface area, central disk diameter and aboral arm length.

Models were conducted in R (Ver. 5.1.13). Model assumptions were examined using the dHARMA package (Hartig 2024). All models met assumptions of normality and homogeneity of variance.

## 3 Results

### 3.1 Fertilisation Assay

For all cross types, fertilisation success significantly increased as sperm concentration increased (Table 1, Figure 2). Cross type had a significant effect on fertilisation success. The degree of change varied from the control As ♂ × As ♀ compared to other crosses, with As ♂ × As ♀ displaying a lower rate of change in fertilisation success with increasing sperm density. Fertilisation success peaked at 10^5^ concentrations in As ♀ × Ab ♂ treatment (~80%), with the As ♀ × As ♂, Ab ♀ × As ♂ and Ab ♀ × Ab ♂ crosses having maximum fertilisation at the 10^6^ sperm concentration (~80%, ~95% and ~80%, respectively). Fertilisation success was seen in low sperm concentrations (10^2^ sperm mL^−1^) in all crosses (15%–25%). Post hoc pairwise analysis indicated that there was no difference in fertilisation success between controls and there was significantly higher fertilisation success in the hybrid crosses (TukeyHSD: As ♂ × As ♀ = Ab ♀ × Ab ♂ < Ab ♀ × As ♂ = As ♀ × Ab ♂).

**TABLE 1:** Interactive binomial generalised linear mixed effects model (GLMM) analysis of fertilisation success data as a function of sperm concentration across crosses in *Acanthaster* cf. *solaris* and *Acanthaster brevispinus* .

Effect	Estimate	SE	*Z* value	*p*
Sperm dilution	1.25622	0.08809	14.260	< 0.001***
*A. brevispinus* ♀ × *A. solaris* ♂	2.08041	0.6290	3.329	< 0.001***
*A. solaris* ♀ × *A. brevispinus* ♂	1.82043	0.60836	2.992	< 0.001***
*A. solaris* ♂ × ♀	1.29644	0.51479	2.518	0.01790*
Sperm dilution: *A. brevispinus* ♀ × *A. solaris* ♂	−0.10032	0.15919	−0.630	0.528565
Sperm dilution: *A. solaris* ♀ × *A. brevispinus* ♂	−0.14632	0.14627	−1.000	0.317141
Sperm dilution: *A. solaris* ♂ × ♀	−0.30334	0.11864	−2.557	0.010563*

**FIGURE 2:**
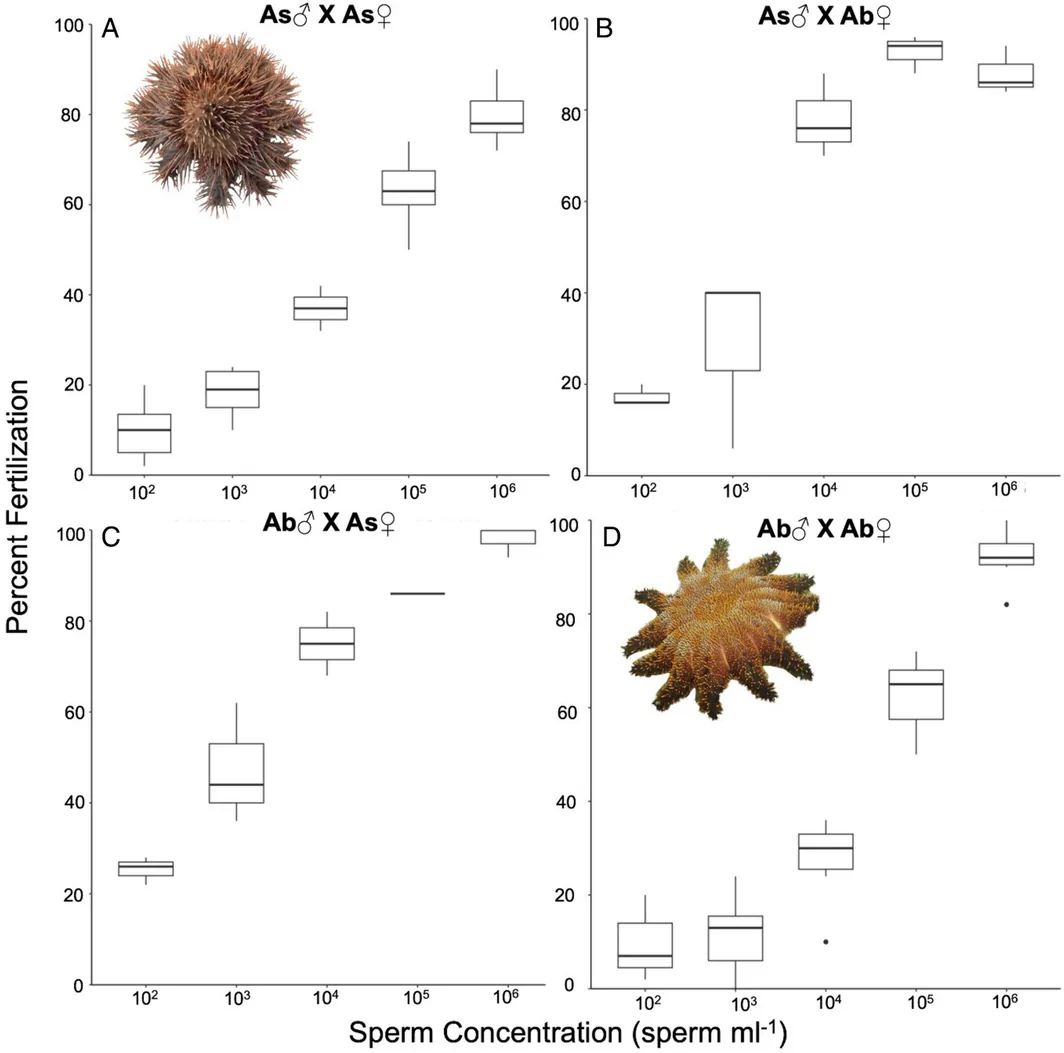
Mean percent fertilisation of conspecific and heterospecific crosses of *Acanthaster* cf. *solaris* (As) and *Acanthaster brevispinus* (Ab) across a range of sperm concentrations (10^2^–10^6^ sperm mL^−1^). (A) As ♂ × As ♀ (*n* = 6); (B) As ♀ × Ab ♂ (*n* = 3); (C) Ab ♀ × As ♂ (*n* = 3); (D) Ab ♂ × Ab ♀ (*n* = 6). Boxplots show median fertilisation success percent (middle horizontal line), the interquartile fertilisation success percent (rectangle) and range (vertical line/dots). Data points represent individual observations.

The diameter of the eggs from females used for fertilisation assays did not differ between the two *Acanthaster* species (*F*_1_ = 1.3964, *p* = 0.3041). The average size of *Acanthaster* cf. *solaris* eggs was 224.28 (SE = 9.77), with *Acanthaster brevispinus* eggs 2% larger with a mean of 229.84 (SE = 9.37) (Table 2).

**TABLE 2:** Unfertilised egg diameter (μm) of *Acanthaster* cf. *solaris* and *Acanthaster brevispinus* of individual females with standard error (SE).

Species	Female	Mean diameter (μm)	SE	Combined mean diameter (μm)	SE
*Acanthaster* cf. *solaris*	1	227.07	5.80		
	2	233.04	6.79	224.28	9.77
	3	221.35	8.36		
	4	218.44	7.31		
*Acanthaster brevispinus*	1	226.58	9.83		
	2	230.96	7.91	229.84	9.37
	3	231.98	9.54		

### 3.2 Larval Morphology and Rearing

Larval width (*F*_3_ = 12.929, *p* ≤ 0.001), surface area (*F*_3_ = 8.3989, *p* ≤ 0.001) and ciliated band length (*F*_3_ = 7.0898, *p* = 0.00132) differed significantly across treatments (Table 3). Larval length was similar across treatments. Tukey's HSD test revealed that As ♀ × Ab ♂ hybrids were larger in width compared to the control As ♀ × As ♂ larvae (Tables 3 and 4). Subsequently, the As ♀ × Ab ♂ hybrids were larger in surface area compared to Ab ♀ × As ♂ hybrids. The hybrids of Ab ♀ × As ♂ had lower ciliated band lengths compared to other treatments. All treatments reached competent brachiolaria settlement stage (Figure 3).

**TABLE 3:** Results of 2‐way ANOVAs of the morphological traits of larval *Acanthaster* cf. *solaris* and *Acanthaster brevispinus* and their hybrid crosses.

Morphological trait	Source	df	*F*‐ratio	*p*	TukeyHSD
Larval length	Treatment	3	1.4746	0.2455	As ♀ × As ♂ = Ab ♀ × As ♂ = As ♀ × Ab ♂ = Ab ♀ × Ab ♂
	Residual	25			
Larval width	Treatment	3	12.929	< 0.001	Ab ♀ × As ♂ = Ab ♀ × Ab ♂ = As ♀ × As ♂ < As ♀ × Ab ♂ = Ab ♀ × As ♂
	Residual	25			
Larval surface area	Treatment	3	8.3989	< 0.001	As ♀ × Ab ♂ > Ab ♀ × As ♂ = Ab ♀ × Ab ♂ = As ♀ × As ♂ < As ♀ × Ab ♂
	Residual	25			
Ciliated band length	Treatment	3	7.0898	0.00132	Ab ♀ × As ♂ < As ♀ × Ab ♂ = Ab ♀ × Ab ♂ = As ♀ × As ♂
	Residual	25			

**TABLE 4:** Average size data of larvae of *Acanthaster* cf. *solaris* and *Acanthaster brevispinus* at bipinnaria (7 days post fertilisation) for individual species and hybrids.

Fertilisation	Length (μm)	Width (μm)	Larval surface area (μm^2^)	Ciliated band length (μm)	Sample size
*Acanthaster* cf. *solaris* × *Acanthaster* cf. *solaris*	714.96 (± 87.73)	467.21 (± 46.90)	235,386 (± 42,422.23)	4019.89 (± 481.68)	10
*Acanthaster brevispinus* × *Acanthaster brevispinus*	683.30 (± 54.59)	516.45 (± 61.79)	238,035.13 (± 35,362.66)	3953.19 (± 258.56)	4
*Acanthaster brevispinus* ♂ × *Acanthaster* cf. *solaris* ♀	731.35 (± 62.58)	600.54 (± 46.32)	290,181.26 (± 27,959.45)	3476.79 (± 732.06)	11
*Acanthaster brevispinus* ♀ × *Acanthaster* cf. *solaris* ♂	643.18 (± 96.32)	530.97 (± 52.44)	190,674.89 (± 46,572.23)	2566.63 (± 275.14)	4

*Note:* Length (μm), width (μm), surface area (μm^2^), ciliated band length (μm). Standard deviation and sample size included with each mean measurement.

**FIGURE 3:**
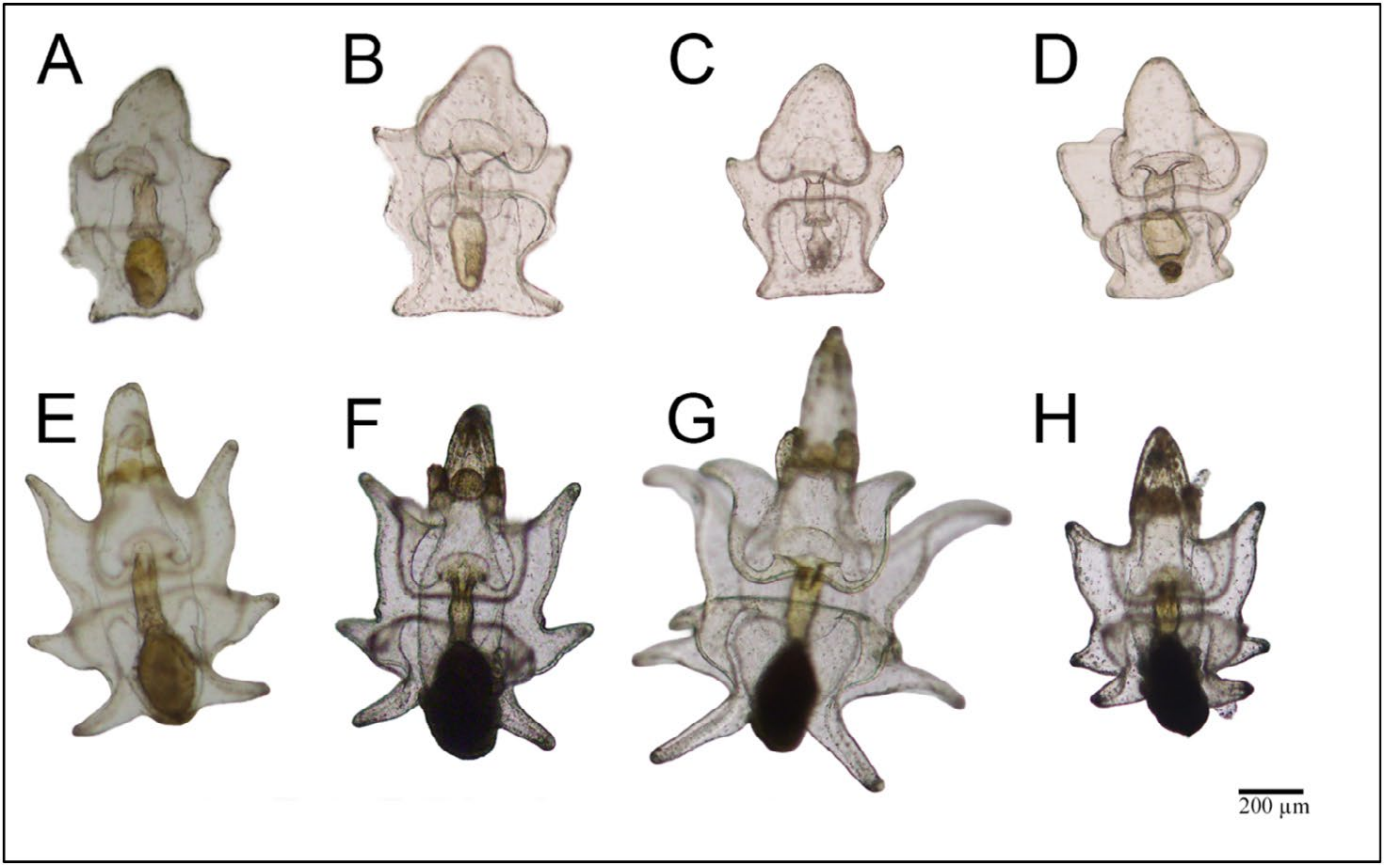
Larvae of *Acanthaster* cf. *solaris* (As), *Acanthaster brevispinus* (Ab) and hybrids of the two species at bipinnaria and brachiolaria stages. (A) As bipinnaria; (B) Ab bipinnaria; (C) Ab ♀ crossed with As ♂ bipinnaria; (D) Ab ♂ × As ♀ bipinnaria; (E) As brachiolaria; (F) Ab brachiolaria; (G) Ab ♀ × As ♂ brachiolaria; (H) Ab ♂ × As ♀ brachiolaria. Scale bar = 200 μm.

### 3.3 Settlement and Juvenile Development

Newly settled larvae attached to *Amphiroa* had a mushroom‐like appearance as the larval body was resorbed into the rudiment (Figure 4). Newly metamorphosed juveniles (~24 h post metamorphosis) had a stellate five‐armed profile (mean diameter 286.13 μm; SE = 56.4 μm, *n* = 10) (Table 5). Juveniles at ~5 days post metamorphosis (dpm) had increased in diameter by approximately 70% with a mean diameter of 415.94 μm (SE = 61.26, *n* = 13). There was minimal growth between 5 dpm and 30 dpm as body profile became more refined. At 78 dpm the juveniles had a mean diameter of 544.2 μm (SE = 150.54, *n* = 12), a 25% increase in size, and began to develop additional arms. As both hybrid juveniles developed, they changed from a stellate sea star to become more central disk dominated in body plan (Figure 5). There was no difference in growth between controls and hybrid juveniles over the developmental period, with consistent growth trends throughout the sampling period (Table 6). At no stage did the hybrid juveniles develop the distinct pink colour trait that is characteristic of *Acanthaster* cf. *solaris* and matches the colour of their coralline algal food and habitat (Figure 5).

**FIGURE 4:**
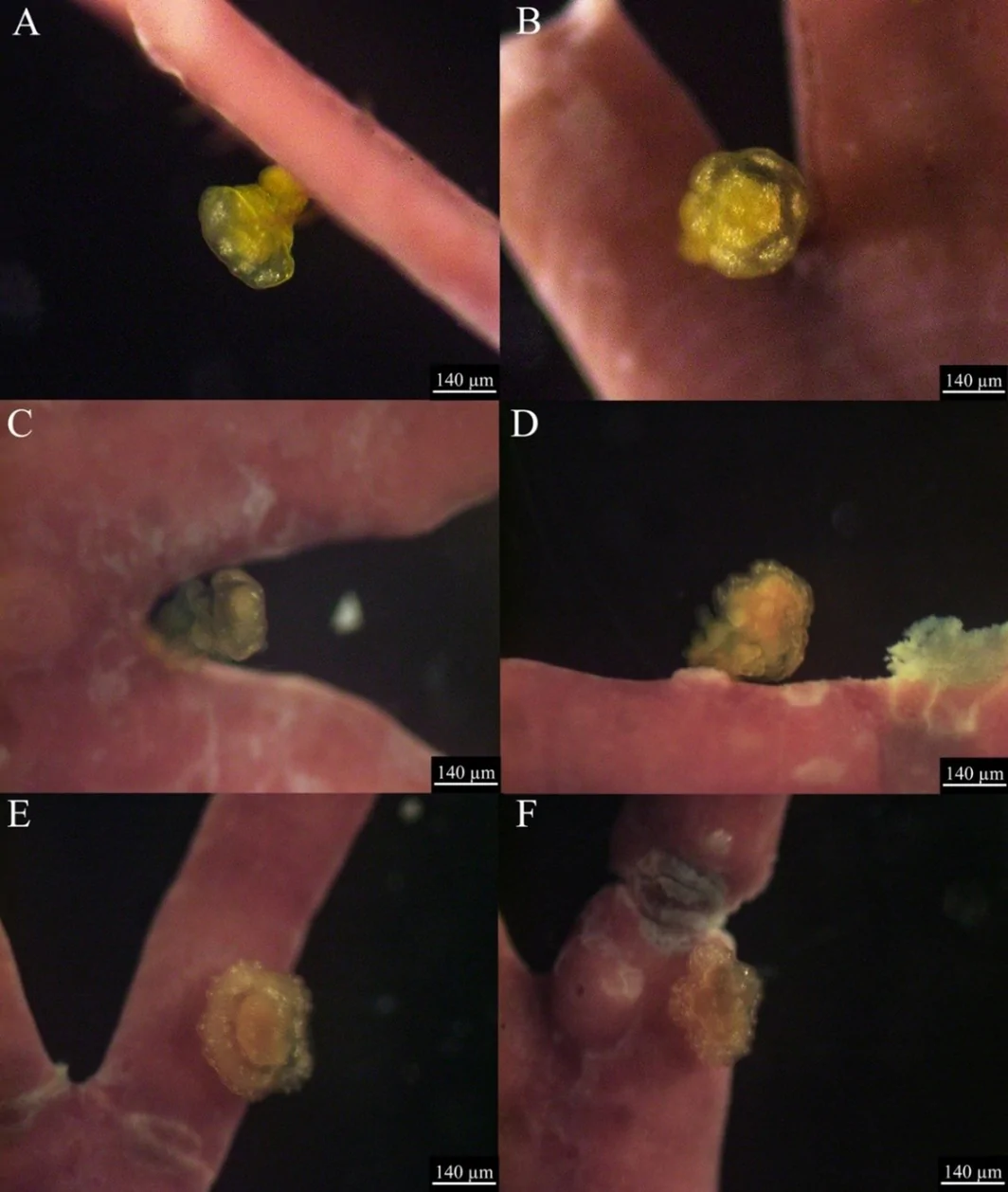
(A, B) Newly attached *Acanthaster* cf. *solaris* larva. (C, D) Newly attached *Acanthaster* cf. *solaris* ♂ and *Acanthaster brevispinus* ♀ hybrid larva. (E, F) Rudiment development in metamorphosing hybrid larvae. Scale bars = 140 μm.

**TABLE 5:** Average size data of both cross‐type juveniles of *Acanthaster* cf. *solaris* and *Acanthaster brevispinus* at different days post settlement for both fertilisation combinations.

Fertilisation combination	Days post settlement	Diameter (μm)	Larval surface area (μm^2^)	Central disk diameter (μm)	Aboral arm length (μm)	Number of arms	Sample size
*Acanthaster brevispinus* ♀ × *Acanthaster solaris* ♂	1	289.44 (± 65.86)	44,450.22 (± 5206.29)	166.44 (± 59.03)	56.37 (± 4.12)	5 (± 0)	7
	5	418.73 (± 51.68)	134,312.12 (± 42,294.26)	253.78 (± 36.64)	112.38 (± 15.07)	5 (± 0)	10
	30	427.81 (± 78.88)	149,479.51 (± 70,317.69)	261.36 (± 54.83)	110.41 (± 27.11)	5 (± 0)	6
	78	499.82 (± 120.04)	198,679.63 (± 90,078.22)	294.89 (± 80.35)	123.95 (± 30.85)	5.29 (± 0.49)	7
	134	763.63 (± 132.23)	464,974.00 (± 154,061.12)	459.63 (± 88.54)	175.06 (± 35.51)	6.5 (± 0.71)	2
	195	872.09 (± 329.57)	634,481.24 (± 418,123.85)	590.35 (± 277.29)	218.30 (± 100.84)	7 (± 1.41)	2
	239	1108.85	957,618.79	816.86	280.77	8	1
*Acanthaster solaris* ♀ × *Acanthaster brevispinus* ♂	1	278.87 (± 33.96)	58,682.65 (± 11,157.16)	189.41 (± 55.89)	55.01 (± 8.64)	5 (± 0)	3
	5	406.64 (± 101.58)	118,127.19 (± 53,926.94)	216.09 (± 48.62)	110.75 (± 40.87)	5 (± 0)	3
	30	461.25 (± 109.50)	168,673.29 (± 63,149.84)	280.52 (± 61.63)	112.40 (± 26.08)	5 (± 0)	5
	78	606.33 (± 180.11)	298,552.24 (± 129,586.35)	376.13 (± 89.08)	151.80 (± 45.80)	5.8 (± 0.61)	5
	134	712.20 (± 137.66)	370,282.22 (± 126,561.23)	437.20 (± 81.24)	166.61 (± 37.30)	6 (± 0.71)	9
	195	856.47 (± 127.36)	557,230.53 (± 161,196.54)	529.58 (± 36.51)	204.58 (± 35.87)	7 (± 0.58)	7
	239	928.20 (± 97.24)	675,327.67 (± 120,958.25)	585.40 (± 36.51)	216.11 (± 29.12)	7.67 (± 1.15)	3

*Note:* Diameter (μm), surface area (μm^2^), central disk diameter (μm), aboral arm length (μm) and number of arms. Standard deviation and sample size included with each mean measurement.

**FIGURE 5:**
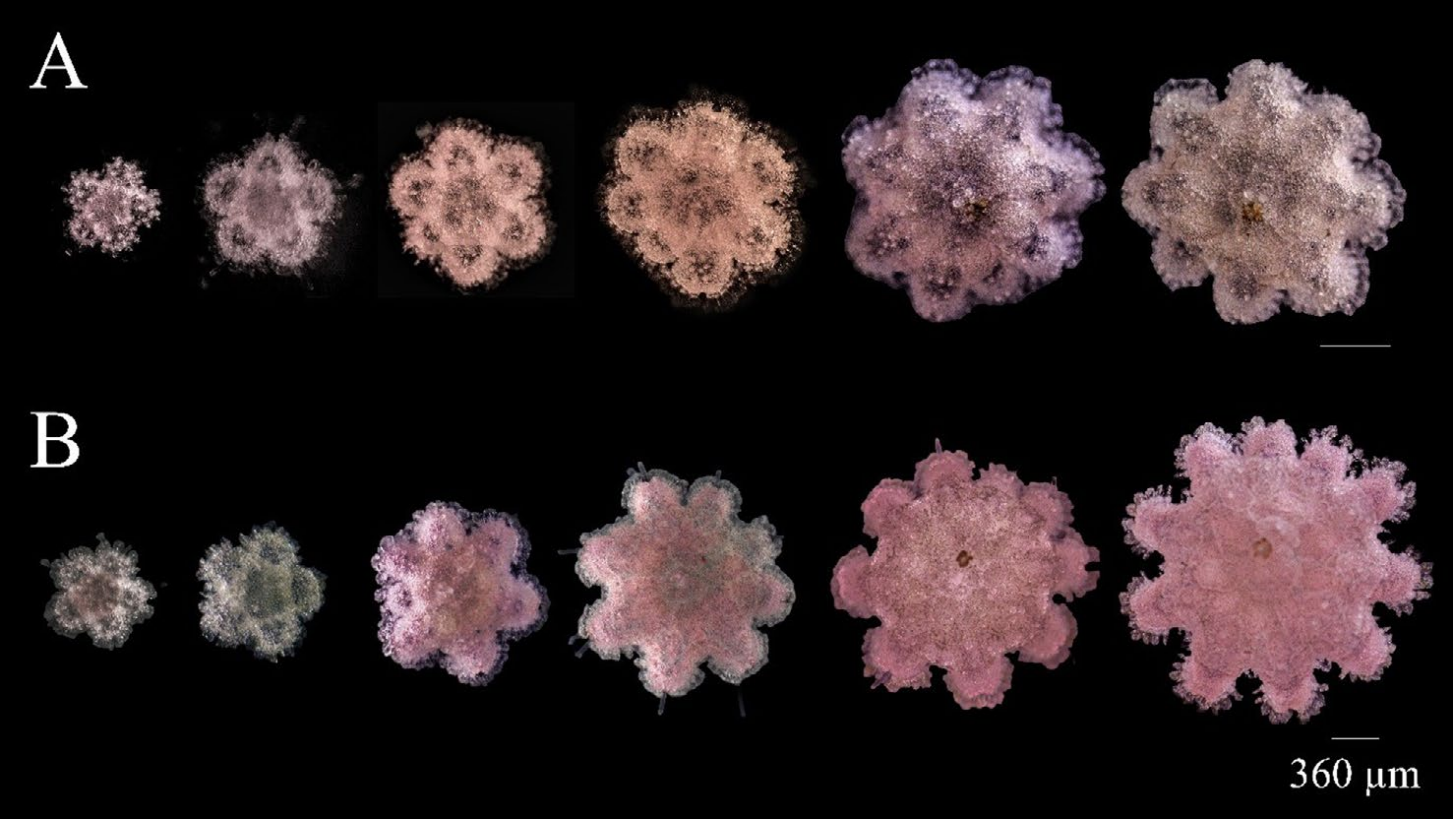
(A) Hybrid juvenile *Acanthaster* cf. *solaris* ♂ × *Acanthaster brevispinus* ♀ at different dps. From left to right: 5, 30, 78, 134, 195 and 239 dps. (B) Juvenile *Acanthaster* cf. *solaris* at different dps. From left to right: 5, 30, 62, 120, 180 and 250 dps. Note the white colour of the oldest hybrid juvenile and the pink colour of *Acanthaster* cf. *solaris*. Scale bars are equal to 360 μm.

**TABLE 6:** Multivariate analysis of variance (MANOVA) using Wilks' test evaluating the difference in growth parameters in juveniles over time (1–79 days post metamorphosis) between hybrid pairs of *Acanthaster* cf. *solaris* and *Acanthaster brevispinus* .

Response	df	Wilks	*F* value	*p*
Treatment	1	0.84953	1.7270	0.1636
Days post metamorphosis	1	0.54924	8.0019	< 0.001***
Treatment × Days post metamorphosis	1	0.89673	1.228	0.3598
Residuals	42			

***p < 0.001.

## 4 Discussion

The redistribution of species due to climate change is causing new species interactions with potentially unexpected consequences, especially when closely related sister taxa are present (Mallet 2007; Pecl et al. 2017). Recent mixed species outbreaks in the subtropics indicate the formation of a secondary contact zone between two coral‐eating *Acanthaster* spp. (Sommer et al. 2025) and the possible formation of a hybrid zone as seen for other congeneric sea star species (Avise et al. 1987; Harrison 1993; Arnold 1997, 2006). Here we show the hybridisation potential and development across a range of sperm conditions to limiting sperm concentration, highlighting the possibility for species introgression. The fertilisation success in hybrid pairings was higher than that of control treatments, indicating the potential for natural hybridisation (Mallet 2007). In addition, as previously reported (Lucas and Jones 1976), the hybrid larvae developed normally to the late juvenile stage for both reciprocal crosses at which time observations ceased. Notably, this shows not only that the gametes are compatible, but also the combined genomes to produce the juvenile, thereby creating the potential for species introgression and hybrid speciation (Pfenning et al. 2016).

Broadcast spawners exhibit species‐specific fertilisation mediated by gamete recognition proteins, typically on the surfaces of sperm and egg cells (Metz et al. 1994; Summers and Hylander 1975; Vacquier and Moy 1977). These proteins contribute to reproductive isolation in broadcast spawners (Biermann 1998). While the two *Acanthaster* spp. are reproductively compatible, their usually contrasting habitats (shallow vs. deep water) provide reproductive isolation through ecological separation. Other factors such as gamete release at different times or specific pheromonal spawning cues may also function in prezygotic isolation (Byrne 1992; Harper and Hart 2005, 2007). However, at this stage there seems to be no reproductive isolation inhibiting the hybridisation of these species (Mallet 2007).

The fertilisation success of the *Acanthaster* cf. *solaris* controls in our study was lower than previously reported (Benzie and Dixon 1994). This difference in fertilisation success may be influenced by gamete fertility with regards to the breeding condition of the two CoTS species. The time of our experiments in February was near the end of the spawning period of *Acanthaster* cf. *solaris* on the Northern GBR where the animals were sourced (Caballes et al. 2021) and this may have influenced fertility (see Benzie and Dixon 1994). While a cross with *A*. cf. *solaris* individuals from SIMP would have been desirable, no *A*. cf. *solaris* were available at the time of the experiment. We acknowledge that we had a low sample size, a limitation due to the opportunistic nature of this study in response to the unexpected appearance of *Acanthaster brevispinus* in SIMP (Sommer et al. 2025) and specimens collected by the culling team. As a result, there was a limited number of reproductively mature individuals available. In addition, as there is no information on the reproductive cycle and spawning season of *Acanthaster brevispinus* we do not know if the timing of our experiment coincided with peak spawning season, although the high rates of fertilisation in the controls indicated high gamete fertility.

The two *Acanthaster* species have similarly sized eggs and so presented a similar target size to sperm. *Acanthaster* species eggs are much larger than those of most sea stars with planktonic larvae where the eggs are generally < 150 μm in diameter (Miner et al. 2005; Caballes et al. 2016). As the egg target size is key to fertilisation kinetics (Levitan 1993), the similar egg size of the two COTS species indicates that their sperm‐egg dynamics will be similar. In previous sea star hybridisation studies, the success of the reciprocal cross was influenced by gamete type such as the egg of one species was more prone to cross fertilise than that of the other species (Harper and Hart 2005; Byrne and Anderson 1994). For instance, in *Asterias* species, some hybrid crosses were more likely due to the sperm being more compatible, resulting in higher fertilisation success even when they were an order of magnitude less abundant (Harper and Hart 2005). However, this was not seen in the hybridisation of *Acanthaster* spp. in our study, where reciprocal crosses had similar fertilisation success. This is likely to be influenced by the similar egg size of the two *Acanthaste*r spp. (Levitan 1993). The hybrid juveniles were similar in growth morphometrics up to 78 days post settlement.

Larval development in the control and hybrid crosses was similar. Although we noted differences in the sizes of larvae of the four crosses, with the hybrid larvae having larger surface areas and shorter ciliated band lengths. However, ciliary band length is a phenotypically plastic trait in sea star larvae in response to food levels (Wolfe et al. 2015). While the cultures started at ~10 larvae per ml, we did not track larval density/mortality over time. Thus, differences in larval size may have been influenced by varying amounts of food available per larva and so apparent differences in the larval ciliary band length would need to be revisited.

The documented morphological distinction between hybrids and *A*. cf. *solaris* juveniles is in the development of spines, with differences occurring at diameters more than 200 mm (Lucas and Jones 1976). Hybrids were shown to have a morphological intermediate spine length comparative to *Acanthaster* spp. (Lucas and Jones 1976), with the intermediate hybrid features of the late hybrid juveniles thought to be more consistent with the *A. brevispinus* condition (Lucas and Jones 1976). We were unable to assess this here as our *A. brevispinus* larvae did not settle while both hybrid pairings settled in the presence of *Amphiroa* spp. As the developmental biology of *A. brevispinus* is understudied and the two species would largely settle and metamorphose in different habitats, settlement cues may differ between the two species.

A key trait in juvenile *A*. cf. *solaris* is the pink pigmentation that they develop, a colour that matches their coralline algal food (Wilson et al. 2025). This pink colour did not develop in the hybrid juveniles generated by both reciprocal crosses up to 239 days post metamorphosis. However, as we did not rear *A. brevispinus* , we do not know if this lack of a pink colour in the juvenile is a feature of this species. This is potentially important in population assessments and management as this trait might be useful to identify juveniles to the species level in the field.

Range edge populations of species typically have lower genetic diversity compared to core populations due to founder effects, genetic drift and isolation (Eckert et al. 2008). Population genetic analyses show that the *Acanthaster* cf. *solaris* in SIMP are genetically connected with populations in the GBR (Nimbs et al. 2025). This connectivity is suggested to be mediated by larvae dispersed from the GBR poleward towards SIMP in the East Australian Current (Condie et al. 2012; James et al. 2002; Nimbs et al. 2025). There are no data on the population genetics of *A. brevispinus* . In secondary contact zones for hybrid viable populations of echinoderms, it can be difficult to determine if hybridisation is occurring through morphometric analysis alone (Zigler et al. 2012; Byrne 1992; Byrne and Anderson 1994; Harper and Hart 2005, 2007). In echinoderms, morphologically intermediate individuals are often F1 hybrids, and when F1 hybrids are not observed it is unclear whether hybridisation is ongoing without conducting genetic analysis (Zigler et al. 2012; Uthicke et al. 2005). If these mixed aggregations of *A*. cf. *solaris* and *A. brevispinus* become more common, genetic analysis may be needed to determine if hybrids occur in nature.

Our results are consistent with previous research showing that F1 hybrids are morphological intermediates of *Acanthaster* spp. up to 2 years post settlement (Lucas and Jones 1976). As morphological intermediates, the hybrids have potential for heterosis in these subtropical reefs (Wang et al. 2024). Notably, hybridisation could facilitate inheritance of thermal resilience due to the contrasting thermal climates in the habitat (e.g., *A. brevispinus* —cooler, *A*. cf. *solaris*—warmer) of the two species (Lamare et al. 2014; Endean 1961). Heterosis of thermal tolerance is well documented in echinoderms for aquaculture purposes (Tian et al. 2024; Han et al. 2019) and in natural hybrids of *Asterias* (Giakoumis et al. 2023). The persistence of cold tolerant obligate corallivores is therefore possible on these high latitude reefs, with potentially profound impacts on vulnerable high‐latitude coral communities as seen in recent outbreak events in Australia and Japan (Yasua et al. 2017; Sommer et al. 2025).

The fertilisation potential and subsequent development between COTS sister taxa identified here indicates the potential for hybridisation in secondary contact zones. This hybridisation can further drive the range expansion or hybrid speciation through reticulate evolution (Bronstein et al. 2019; Pfenning et al. 2016). The inheritance of an intermediate thermal range in hybrids poses an inherent risk to vulnerable high latitude coral communities due to the introduction of cold tolerant obligate corallivores. The lack of observable reproductive isolation between the two species highlights the need to understand the reproductive and developmental biology of *A. brevispinus* . Further research into the population genetics of the two species at secondary contact zones is needed to determine the potential for species introgression.

## Author Contributions

**Ronan T. Hill:** conceptualization (equal), data curation (lead), formal analysis (lead), investigation (equal), methodology (equal), project administration (equal), visualization (lead), writing – original draft (lead). **Matthew Clements:** data curation (supporting), formal analysis (supporting), investigation (supporting), methodology (supporting), project administration (supporting), writing – review and editing (supporting). **Monique Webb:** data curation (equal), investigation (supporting), methodology (supporting), project administration (supporting). **Brigitte Sommer:** conceptualization (supporting), funding acquisition (lead), resources (lead), supervision (supporting). **Maria Byrne:** conceptualization (lead), data curation (equal), formal analysis (supporting), funding acquisition (equal), investigation (equal), methodology (lead), project administration (equal), resources (lead), supervision (lead), visualization (supporting), writing – review and editing (lead).

## Funding

This work was supported by Australian Research Council Discovery Early Career Research Award (DE230100141).

## Conflicts of Interest

The authors declare no conflicts of interest.

## Data Availability

The data in this manuscript are publicly available in the University of Sydney repository via https://doi.org/10.25910/wca3‐2k49.
